# Report of Columbia Hospital for Women

**Published:** 1873-11

**Authors:** 


*Report of Columbia Hospital for Women and Lying-in Asylum, Washington, D. C.* By J. Harry Thompson, A. M., M. D., Surgeon-in-Chief. Washington: Government Printing Office, 1873.

The Columbia Hospital for Women was established in 1866, and since that period has been in active operation. The volume under consideration is the first report of the institution, and reflects much credit upon the attending physician.

Art I., on Lacerated Perineum, is an account of thirty-four cases operated on by Dr. J. H. Thompson, These cases have been selected out of the fifty-three cases treated as comprising all the varieties and complications of this accident. The article is a good one, and shows much care in its preparation. It is followed by one on Vesico-Vaginal and Recto-Vaginal Fistula, which is an admirable paper on the subject. A paper on diseases and displacements of the uterus is one which will be read with as much interest and profit as any in the Report. A consideration of Pelvic Celulites and of Diseases of the Rectum concludes this part of the work, which is from the pen of Dr. Thompson, and is made up of several other very interesting papers, which space will not allow us to mention. The appendix is composed of Reports from the Dispensary connected with Hospital. The reports on Diseases of Women by Dr. F. H. Ashford, on the Diseases of Children by Dr. Samuel C. Busey, and on Diseases of the Eye and Ear by Dr. D. W. Prentiss, are well made, and add much to the interest of the volume. The staff of the Hospital have exercised a commendable amount of care and labor in the preparation of their report and it is to be hoped that they will follow up the work so well commenced.

The report is illustrated by several engravings, which are, as a general thing, well made. Some, however, could have been much improved. The profession are under many obligations of Dr. Thompson for his contribution to its literature.

